# Evaluation of market risk and resource allocation ability of green credit business by deep learning under internet of things

**DOI:** 10.1371/journal.pone.0266674

**Published:** 2022-04-07

**Authors:** Fan He, Meitao Wang, Peng Zhou

**Affiliations:** 1 School of Accounting, Chongqing Technology and Business University, Chongqing, China; 2 Budgetary Appraisal Center, Ministry of Finance of the People’s Republic of China, Beijing, China; 3 Center of Engineering and Construction Service, Ministry of Agriculture and Rural Affairs of the People’s Republic of China, Beijing, China; The Bucharest University of Economic Studies, ROMANIA

## Abstract

The research expects to evaluate the capital market risk and resource allocation ability of green credit business exploration based on neural network algorithm by deep learning in the context of the Internet of things, increase the funds flowing to green environmental protection industry, accelerate the development of real economy and stabilize China’s market economy. On the basis of previous studies, the research takes the credit business in the capital market as the research object, and improves the ability of resource allocation by optimizing the financial transaction structure. On this basis, through comparative analysis, the grey system model is implemented. back propagation neural network model under deep learning is used to evaluate the capital market risk of green credit business exploration, and the data of different provinces in China from 2009 to 2019 are taken as an example to verify. The model is used to measure the relationship between green credit business and industrial structure. Additionally, it also analyzes the main factors affecting the efficiency of green credit. The results show that green credit mainly affects the industrial structure through enterprise capital and financing channels. China’s overall green credit adjustment has had a significant upgrading effect on the industrial structure. The impact of green credit on industrial structure adjustment is different in the east, middle, and west regions. Optimizing the project capital structure, promoting seasonal financial transformation, setting up the function of innovation platform, and improving the internal governance structure of enterprises can improve financing efficiency and realize green and sustainable economic development in the future. The research results can provide a theoretical basis for the green development of China’s financial market and the application of deep learning neural network algorithm under the background of Internet of things.

## 1. Introduction

With the rapid growth of China’s economy in recent years, the contradiction between China’s resource and environmental carrying capacity and the people’s growing living needs has become increasingly prominent. Traditional development is based on high consumption and high emission models; such a development method at the expense of the environment has brought short-term rapid development to China’s economy [1]. According to statistics, China’s total real estate loans have accounted for about 29% of all loan balances, very similar to the real estate bubble in Japan, which can bring serious systemic financial risks [2]. In addition, many capital and government regulatory agencies have given the green light to policies with the rapid development of Chinese internet companies to promote the development of the internet industry and ensure the vitality of Chinese companies [3]. The financial market has been fluctuating in recent years; consequently, the integration of a large number of internet companies and the financial market has become a trend. The most typical example is Alipay. At the end of 2020, Alipay was supposed to be listed on the world’s largest IPO (Initial Public Offering). However, Alipay’s business is mostly credit services, realizing the capital credit service of 800 million yuan to 100 billion yuan through the loopholes in the law and supervision [4]. On the eve of the listing, Alipay was collectively stopped by several major regulatory authorities in China. The reason is that Alipay has huge systemic risks. This incident has become the most important event in the financial world this year, revealing that the regulators have noticed the development trend of malformation and speculation in China’s financial market [5]. Despite the short-term high returns, this mode is based on all of China’s economy, which is an unsustainable financial development method [6]. Green credit refers to commercial banks and other financial institutions that provide loan support and preferential interest rates to companies that develop and utilize new energy, companies engaged in circular economy production and manufacturing, and ecological agricultural companies following the environmental economic policies and environmental protection policies specified by the government [7]. Internet finance and real estate bubbles in China’s finance have become prominent issues in the financial sector. How to achieve the development of the green credit business has become a scientific problem that needs to be solved urgently.

Since the Opinions on Implementing Environmental Protection Policies and Regulations to Prevent Credit Risks was promulgated in 2007, guidance, regulations, and development policies of green credit business have been introduced one after another. Green credit has experienced a period of steady development, and its influence on the industrial structure has gradually increased. Chinese banks also regard green credit as an important opportunity for their future development [8]. However, due to the late start and short duration of research on green credit worldwide, the currently published investigations on the influence of green credit on industrial structure transformation and upgrading are scarce. To promote the adjustment of industrial structure and achieve the sustainable development of economy, ecology, and society, the Ministry of Ecology and Environment of the People’s Republic of China has successively issued “Green Credit,” “Green Insurance,” and “Green Securities” policies in cooperation with the People’s Bank of China, China Banking Regulatory Commission, China Insurance Regulatory Commission, and China Securities Regulatory Commission [9]. Regarding the current implementation, the “Green Credit” policy is in the initial stage of practice, while the “Green Insurance” and “Green Securities” policies are in the embryonic stage. Green funds supporting the development of green industries and the green transformation of traditional industries have begun to be issued, and exchanges of carbon emission have been established successively [10]. The green credit policy applies financial leverage to the legalization of environmental protection, curbs the blind expansion and disorderly development of high-energy-consuming and high-polluting industries, achieves energy conservation and emission reduction goals, and supports new energy and the development of the environmental protection industry, and finally, adjusts the industrial structure [11]. As an adjustment tool of the financial market, the optimized transaction structure and resource allocation methods have been discussed in various reports; however, few considerations are put on credit business currently.

Therefore, the credit business is taken as the research object. Based on previous research, the financial transaction structure and resource allocation capability are optimized using case analysis; combined with the problems of China’s financial industry development, the financing problems of enterprises in the financial market are analyzed. Moreover, specific data are used as examples, and an analysis model is constructed to explore the foremost factors affecting enterprises’ financing and platform credit efficiency, in an effort to put forward some ideas for the research on how to make China’s financial capital flow to the real economy. The innovative points are: (1) a set of independent and complete theoretical views has been established based on economics, finance, and investment theory. (2) The prerequisites for researching investment and financing, that is, the creation of financial asset credit and smooth transactions, are emphasized from the perspective of financing. (3) Based on dual information, the market supervision mechanism has been introduced to supplement the financial development structure. Finally, the performance of the model is verified by experiments, to provide experimental direction for the green development and improvement of intelligent performance of China’s financial market.

There are five sections in total. The first section is an introduction, in which the importance of researching green credit business is put forward, and the principal research ideas are determined. The second section is a literature review, in which the research framework is clarified based on the analysis of green credit and the financial industry development. The third section explains the research methodology, in which the financial transaction structural optimization and green resource allocation design are clarified, the data source and analysis process are explained, and the gray system model is constructed. The fourth section presents the results and discussions, including the case analysis of the proposed model, the performance advantages of the model, and the comparative analysis with some state of the art methods. The fifth section introduces the research conclusion, including the key results and actual contributions, limitations, and prospects for the future.

## 2. Literature review

### 2.1. Research on green credit

Research on green credit focuses on the issue of sustainable development. It is necessary to understand the correlation between economic development and industrial structure adjustment before putting forward a series of sustainable development theories of industrial structure. Liu et al. (2017) established a general equilibrium model of computable finance to quantitatively calculate the system effect of the green credit policy. They found that the green credit policy was effective in restraining investment in energy-intensive industries but less effective in adjusting industrial production structure [12]. Ghisellini et al. (2018) deeply investigated the relationship between financial innovation and industrial structure. They pointed out that the purpose of exploring environmental finance was to penetrate the development concept of environmental protection throughout the entire environmental protection industry and achieve sustainable economic development [13]. He et al. (2019) constructed a threshold effect model and investigated the nonlinear relationship between renewable energy investment and green economic development. From the perspective of green credit, the impact of renewable energy investment on the green economy development index was first promotive, then restrictive, and went back to promotive again. Increasing the expenditures of environmental pollution control and adjusting the industrial structure were conducive to improving the green economic development index [14]. Xiong et al. (2019) combined the output slack measurement model to evaluate the efficiency of green credit at the provincial and departmental levels in China from 2010 to 2016. They found that different provinces had large differences in green credit [15]. Guo et al. (2020) used panel data from 2003 to 2016 to estimate the green development efficiency of 34 cities in China based on the SBM-DEA model. Whether industrial agglomeration promoted or hindered the efficiency of green development was determined. The results found that the level of economic development had the most significant positive impact on green development, while environmental regulations had the greatest impact on green development [16]. The above research reveals that unlike traditional financing methods, environmental finance creates green finance tools to guide the development of alternative energy sources and control the advancement of environmental pollution projects, thereby achieving industrial structural optimization and upgrading.

### 2.2. Development of green finance industry

Green finance means that the financial sector regards environmental protection as a basic policy. Potential environmental impacts must be considered in investment and financing decisions, and the potential returns, risks, and costs of environmental conditions must be integrated into the daily financial business. Moreover, attention should be paid to ecological environment protection and environmental pollution treatment in financial business activities to promote the sustainable development of society through the guidance of social-economic resources [17]. There are lots of reports on green finance. Taghizadeh and Yoshino (2019) proposed to establish a green credit guarantee plan and return the tax revenue originally generated by the spillover effect of green energy supply to investors. Such a plan could reduce the risk of green finance and increase the rate of return of green energy projects [18]. Considering the evolving role of green finance, Khan et al. (2021) quantified green finance as "climate mitigation finance" and investigated its impact on the ecological footprint of 26 economies in Asia. The final empirical results showed that green finance reduced the ecological footprint and seemed to be environment-friendly [19]. Cui et al. (2019) pointed out that green development of finance could enhance innovation ability and green economic transformation, help people deal with the challenges of climate change, ecological crisis, and energy security, and realize the sustainable and balanced development of green finance [20]. Zhou et al. (2020) applied the global Principal Component Analysis (PCA) to formulate the green finance development index and constructed a model describing the impact of green finance on economic development. The results showed that the development of green finance had an important role in promoting economic development [21]. He and Yan (2020) believed that the improvement of traditional industries and the rise of green industries were inseparable from the development of green finance, which could promote the growth of the national economy and was the only way for China’s development [22].

In conclusion, the research of the above scholars reveals that scholars in relevant fields mainly study the structural adjustment and intelligent development of the financial industry. But there are few reports on the industrial structure transformation and upgrading of green credit related issues and the application of intelligent algorithms. Additionally, most of the research work is still in the stage of theoretical analysis, and there is a lack of empirical analysis of regional data in China. Therefore, an analytical model is proposed to verify the relationship between green credit business and the change of China’s industrial structure. The exploration aims to finally optimize the financial transaction structure and resource allocation, and provide a better theoretical path for the intelligent and green development of China’s financial field.

## 3. Research methodology

### 3.1. Gray relational analysis

As a part of the portfolio of green finance, green credit can adjust the capital adjustment, act as a platform for both capital demand and surplus, and also serves as a tool for national policy control, leading the upgrading of different industrial structures. The green credit business can be analyzed from the perspective of industrial structure. According to the previous research, the Gray Relational Analysis (GRA) model is employed to measure the industry under the green credit [23]. The correlation can be determined by analyzing the geometric similarity in different sequences. The specific calculation steps are as follows. First, the original reference sequence and the comparison sequence need to be clarified. The reference sequence is expressed as:

$$X_{0}=\left\{X_{0}(1),X_{0}(2),\ldots ,X_{0}(n)\right\}$$ (1)

The comparison sequence can be expressed as:

$$X_{i}=\left\{X_{i}(1),X_{i}(2),\ldots ,X_{i}(n)\right\}$$ (2)

In (1) and (2), *n = 1*, *2*, *…*, *k*, and *i = 1*, *2*, *…*. Then the above sequence undergoes dimensionless processing to eliminate its dimension of quantity. Thus, each sequence is converted into a comparable form. The specific calculation process is as follows:

$$Y_{i}(n)=\frac{X_{i}(n)}{\frac{1}{k}\sum_{n=1}^{k}X_{i}(n)}n=1,2,\ldots ,k;i=1,2,\ldots .$$ (3)

Then the difference between the normalized reference sequence *Y*_*0*_*(n)* and the normalized comparison sequence *Y*_*i*_*(n)* is calculated:

$$\Delta i(n)=\left|Y_{0}(n)-Y_{i}(n)\right|n=1,2,\ldots ,k;i=1,2,\ldots .$$ (4)

$$\Delta i=(\Delta_{i}(1),\Delta_{i}(2),\ldots ,\Delta_{i}(n))i=1,2,\ldots .$$ (5)

Finally, the correlation coefficient and correlation degree can be obtained. The correlation coefficient is described as:

$$\gamma (Y_{0}(n),Y_{i}(n))=\frac{\Delta i(min)+\rho \Delta i(min)}{\Delta i(n)+\rho \Delta i(max)}n=1,2,\ldots ,k;i=1,2,\ldots$$ (6)

where:

Δi(min)=min(Δi(1),Δi(2),…,Δi(n)) (7)

$$\gamma (Y_{0}\Delta i(max)=max(\Delta i(1),\Delta i(2),\ldots ,\Delta i(n))$$ (8)

The resolution coefficient *ρ* = 0.5. The original data are processed via the above steps and substituted into the correlation equation to calculate the gray correlation degree.

### 3.2. Variables and model construction

According to a research on green credit [24], quantitative analysis is performed using the proportion of green credit line in the total loan line of financial institutions. According to China’s statistical method, green credit is identified as a loan to support environmental protection projects and emerging environmental protection industries. The sampling time for analysis using the current green credit balance indicator is relatively short and cannot accurately reflect the differences between various variables. Therefore, referring to [25], the ratio of energy conservation and environmental protection project loans to the total loan line in the *Social Responsibility Announcement* released by the Bank of China are employed to express the financial interrelations ratio. As for the quantitative method of industrial structure, measurements are performed according to the proportion of different output value in GDP (Gross Domestic Product), where the proportion of the primary industry is recorded as AGR, the proportion of the secondary industry is recorded as IGR, and the proportion of the tertiary industry is recorded as SGR. The details about variable definition are presented in Table 1.

**Table 1:** Definition of variables.

Variable	Definition	Symbol
Financial interrelations ratio	Loan amount of energy conservation and environmental protection projects /total loan amount	GLR
Primary industry ratio	Value-added of primary industry /GDP	AGR
Secondary industry ratio	Value-added of secondary industry /GDP	IGR
Tertiary industry ratio	Value-added of tertiary industry /GDP	SGR

Data from 2009 to 2019 are selected for modeling analysis. Moreover, transformation and upgrading of the industrial structure are lagging; that is, the industrial transformation and upgrading of the previous period will have an impact on the current period. Therefore, lagging variables are introduced to build a dynamic model. to ensure the accuracy and reliability of the model, the lagging period (*ISR*_*i*,*t-1*_) variables, including the industrial structural optimization rate, are introduced. The DPD (Dynamic Panel Data) model is adopted, and the provincial panel data is selected for estimation. The basic measurement model is set as follows:

$$ISR_{i,t}=\alpha_{1}ISR_{i,t}+\alpha_{2}GLR_{t}+\alpha_{3}FIR_{i,t}+\alpha_{4}FIPR_{i,t}+\alpha_{5}CPIV_{i,t}+\mu_{i,t}$$ (9)

In (9), *i = 1*, *2*, *3*,*…*, *N* represents the number of sections, *t = 1*, *2*, *3*,*…*, *N* represents a different year, *μ*_*i*,*t*_ is the disturbance items that change with the individual and time, *ISR*_*i*,*t*_ and *ISR*_*i*,*t-1*_ refer to the industrial structure optimization rate of the *i*-th province in years *t* and *t-1*, respectively, *GLR*_*t*_ denotes the financial interrelations ratio in year *t*, the bank characteristic variable’s *GLR*_*t*_ refers to the financial interrelations ratio of the *i*-th province in year *t*, *FIPR*_*i*,*t*_ stands for the financial industry product ratio of the *i*-th province in year *t*, and *CPIV*_*i*,*t*_ describes the degree of central government intervention in regional credit of the *i*-th province in year *t*.

Finally, the industrial structure optimization rate is regarded as the explained variable, the financial interrelations ratio is the explanatory variable, and the financial interrelations ratio, financial industry product ratio, and government credit intervention are regarded as control variables [26]. The financial interrelations ratio measures the proportion of the green credit line in the total loans of financial institutions. Specifically, the variables are defined in Table 2.

**Table 2:** Definition of variables.

	Variable	Definition	Symbol
Explained variable	Industrial structural optimization rate	Sum of the output value of secondary industry and tertiary industry /regional GDP	ISR
Explanatory variable	Financial interrelations ratio	Loan amount of energy conservation and environmental protection projects /total loan amount	GLR
Control variables	Financial interrelation ratio	Sum of deposit and loan balances of regional financial institutions /regional GDP	FIR
	Financial industry product ratio	Value-added of regional financial industry /regional GDP	FIPR
	Government credit intervention	Deposit balance of regional financial institution /loan balance of regional financial institution	CPIV

### 3.3. Structural optimization of transaction

**Fig 1:**
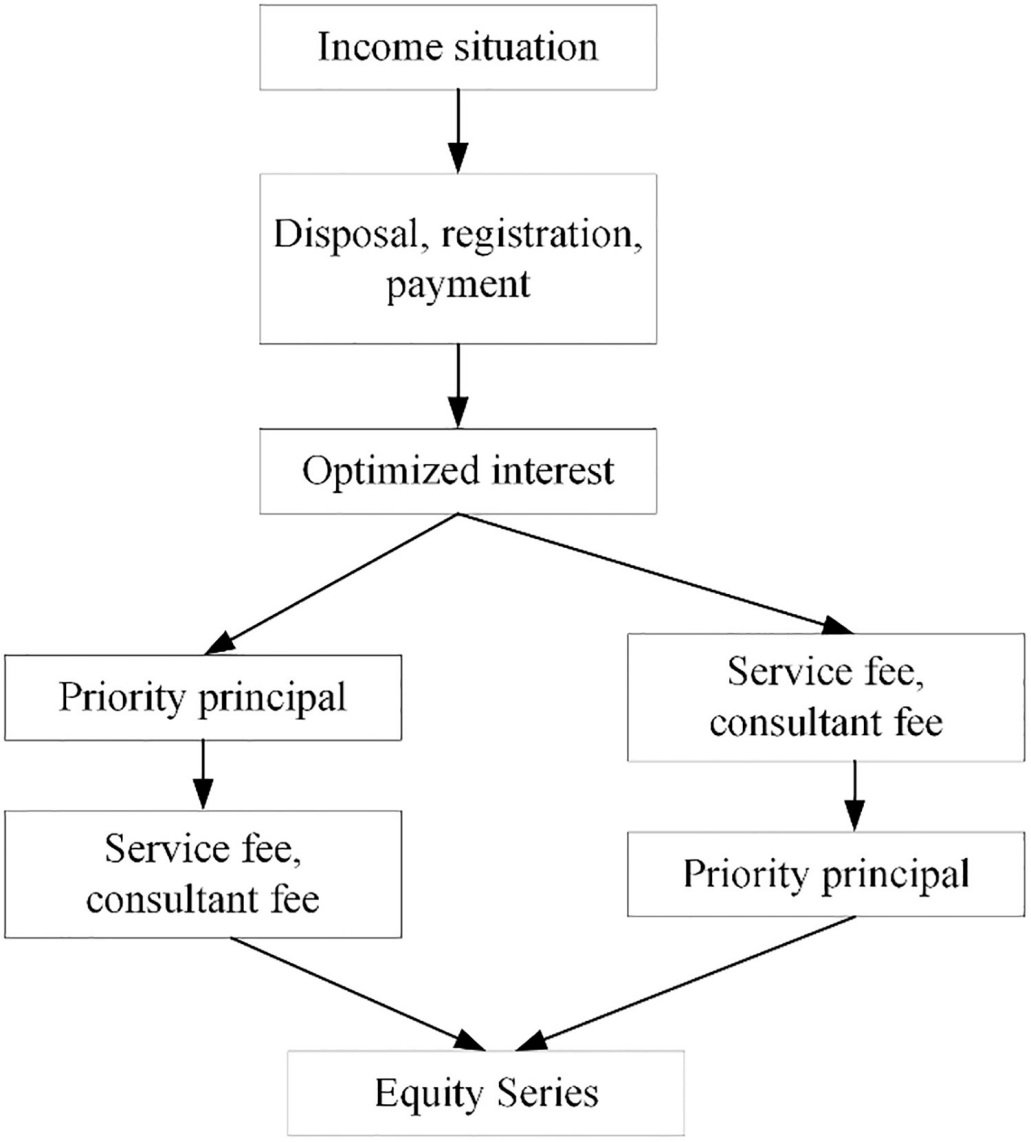
Structural optimization of financial transaction.

Non-performing assets often appear in the crediting process. Although these assets have already been calculated in the risks of the enterprises, the existing risks are often ignored because of the performance in the actual process. The rough structural stratification of China cannot meet the diversified investment needs of investors with different risk preferences. Thus, it is necessary to optimize the transaction structure of the green credit process. The specific optimization plan is presented in Fig 1. The strict stratification of the structure is finer and meets the different investment return requirements of investors with different risk preferences.

### Model performance verification

The financial correlation rate and the financial development level of the model are explained using the method of overall and regional financial analysis. According to the general practice of scholars, the ratio of the sum of financial institutions’ deposit and loan balances in various regions to the GDP of each region is used to express the scale of regional financial development [27]. The financial industry product ratio is adopted to explain the status and development level of the financial industry. The relative scale of the financial industry is calculated by the ratio of the value-added of the regional financial industry to the regional GDP. Government credit intervention reflects the degree of central government intervention in regional credit, expressed as the ratio of deposits and loan balances of regional financial institutions. The data comes from the *China Financial Yearbook* and *China Statistical Yearbook*, including the inter-provincial panel data of 31 provinces in China from 2009 to 2019. The deposit balances, loan balances, value-added of secondary industry and tertiary industry, and GDP data of financial institutions in provinces, municipalities directly under the Central Government, and ethnic minority autonomous regions come from the China Statistical Yearbook, the website of the National Bureau of Statistics, and the websites of the statistical bureaus of each region. Data of loans for energy conservation and environmental protection projects come from the Social Responsibility Announcement released by the Bank of China. The research regions are divided into the eastern region, the central region, and the western region according to Chinese national standards to understand the differences between different regions.

## 4. Results and discussions

### 4.1. Descriptive analysis

**Fig 2:**
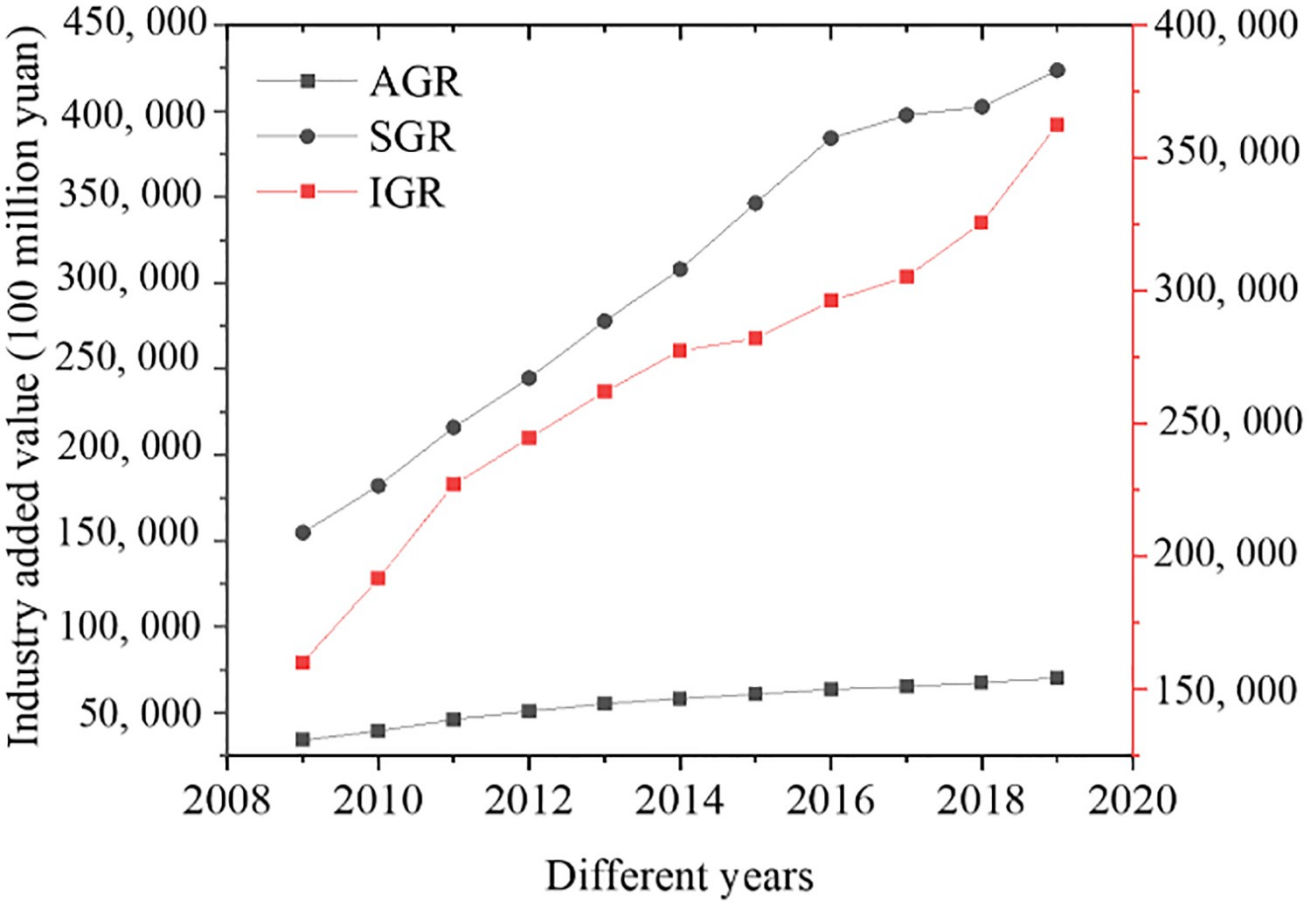
The proportion of the three industries changing over time.

**Fig 3:**
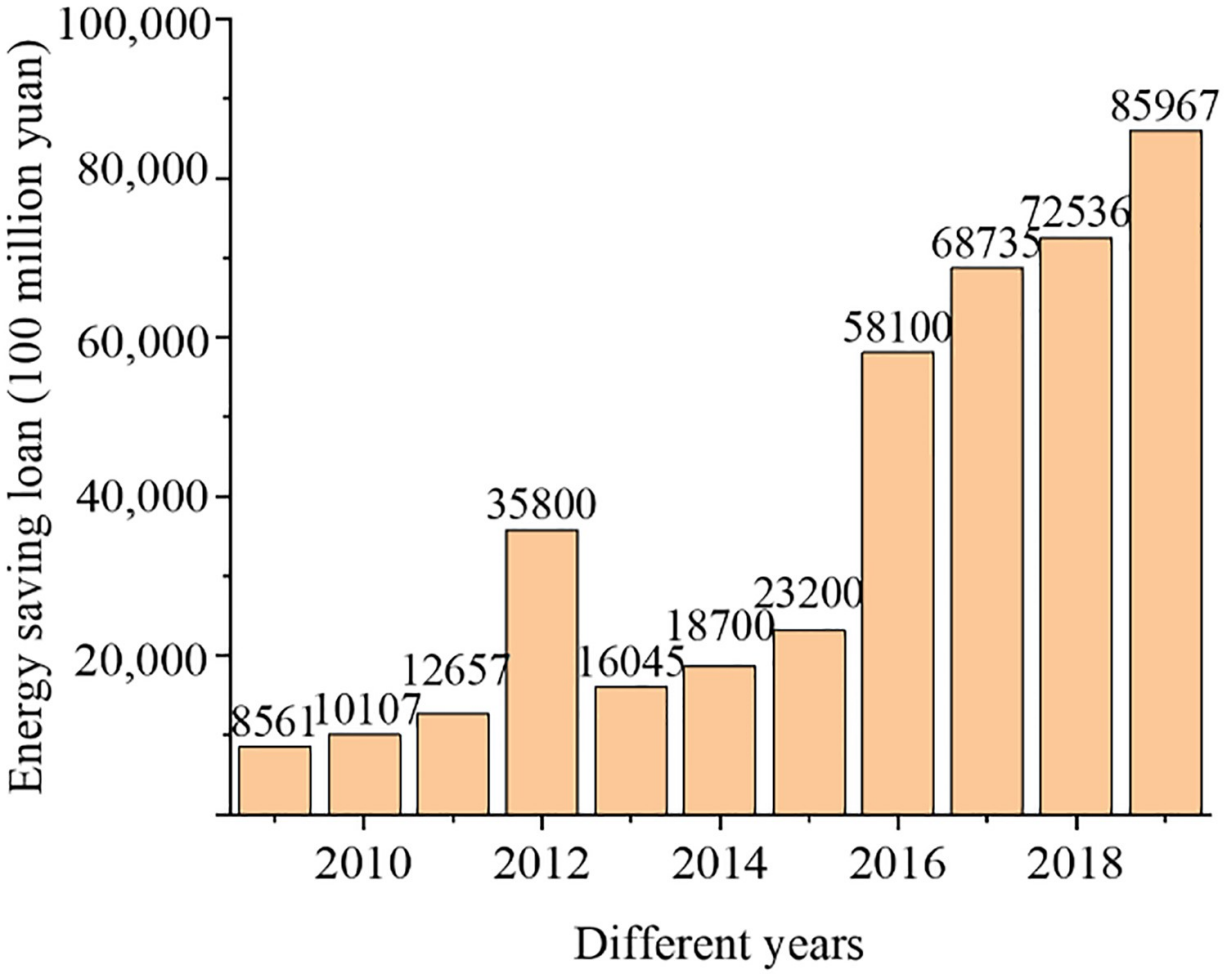
Changes in environmental protection loans over time.

Fig 2 presents the changes in the proportions of the three industries over time, and Fig 3 reveals the changes over time in environmental protection loans. Over time, the proportions of different industries have been increasing. Among them, the primary industry ratio has the smallest increase, the secondary industry ratio tends to stabilize, while the tertiary industry ratio fluctuates, which has slightly decreased in both 2011 and 2015 with an overall trend of increasing. The scale of credit in the field of energy conservation and environmental protection loans is increasing continuously. A sharp drop appeared in 2013; afterward, it starts to increase gradually since 2016. This result owes to the government’s policy support for environmental protection industries.

### 4.2. Analysis of optimization results

**Fig 4:**
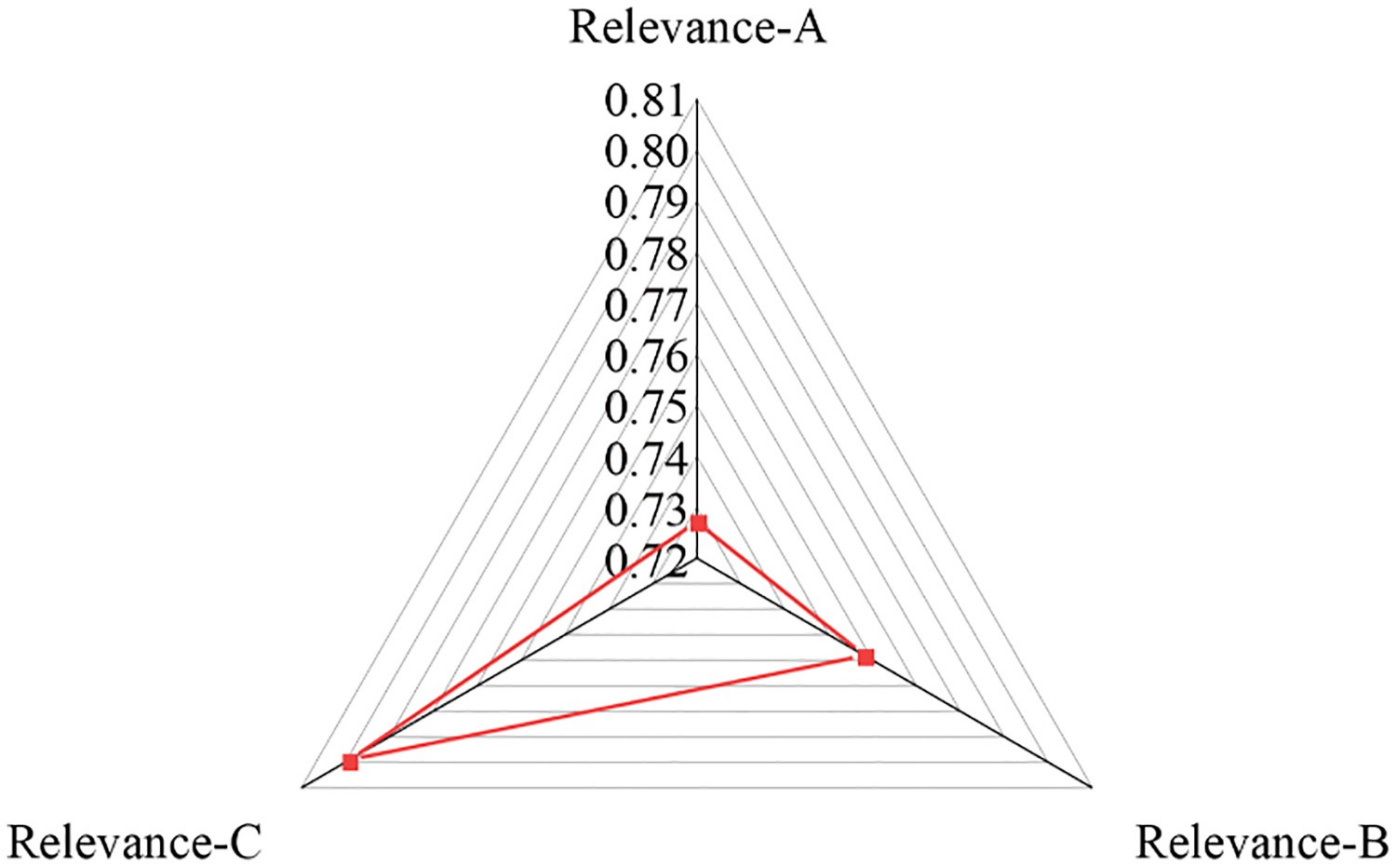
GRA results.

As shown in Fig 4, the tertiary industry occupies the largest proportion, while the primary industry accounts for the smallest, indicating that the gray relation between the financial interrelations ratio and the proportion of tertiary industry in GDP is the largest. This result further illustrates that China’s financial interrelations ratio and the proportions of various industries in its GDP have geometric similarities. However, the gaps between the calculated gray correlation degrees reveal that the change in the sequence of financial interrelations ratio shares the uttermost synergy with the tertiary industry’s proportion sequence, followed by its synergy with the secondary industry’s proportion sequence. When green credit is implemented more extensively, the tertiary industry will benefit the most, followed by the secondary industry; that is, the industrial structures have been optimized and adjusted thrice.

### 4.3. Overall estimation results

**Fig 5:**
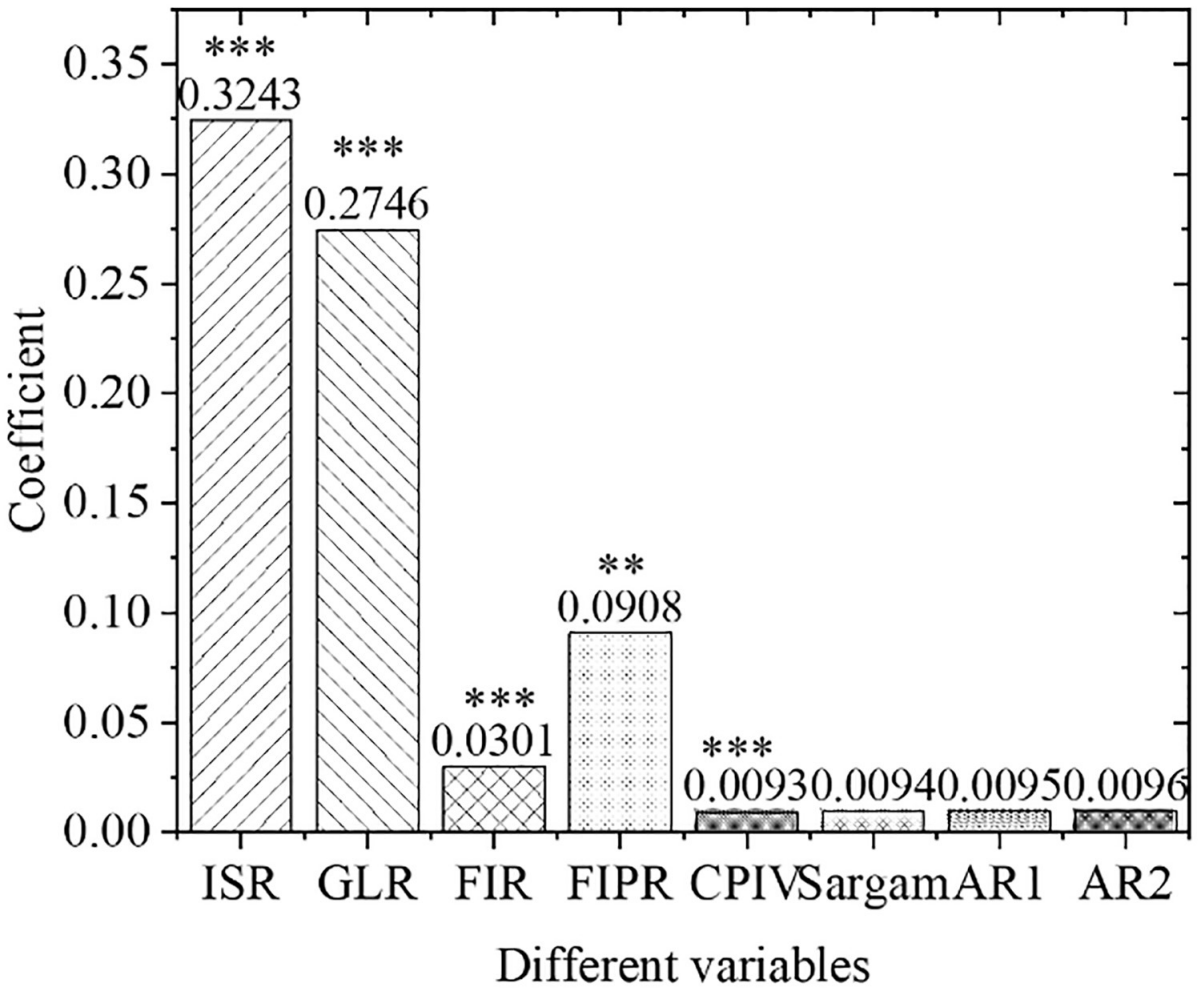
Estimation results of overall empirical panel coefficient. Note: *, **, and *** represent the significance level of 10%, 5%, and 1%, respectively, the same below.

**Fig 6:**
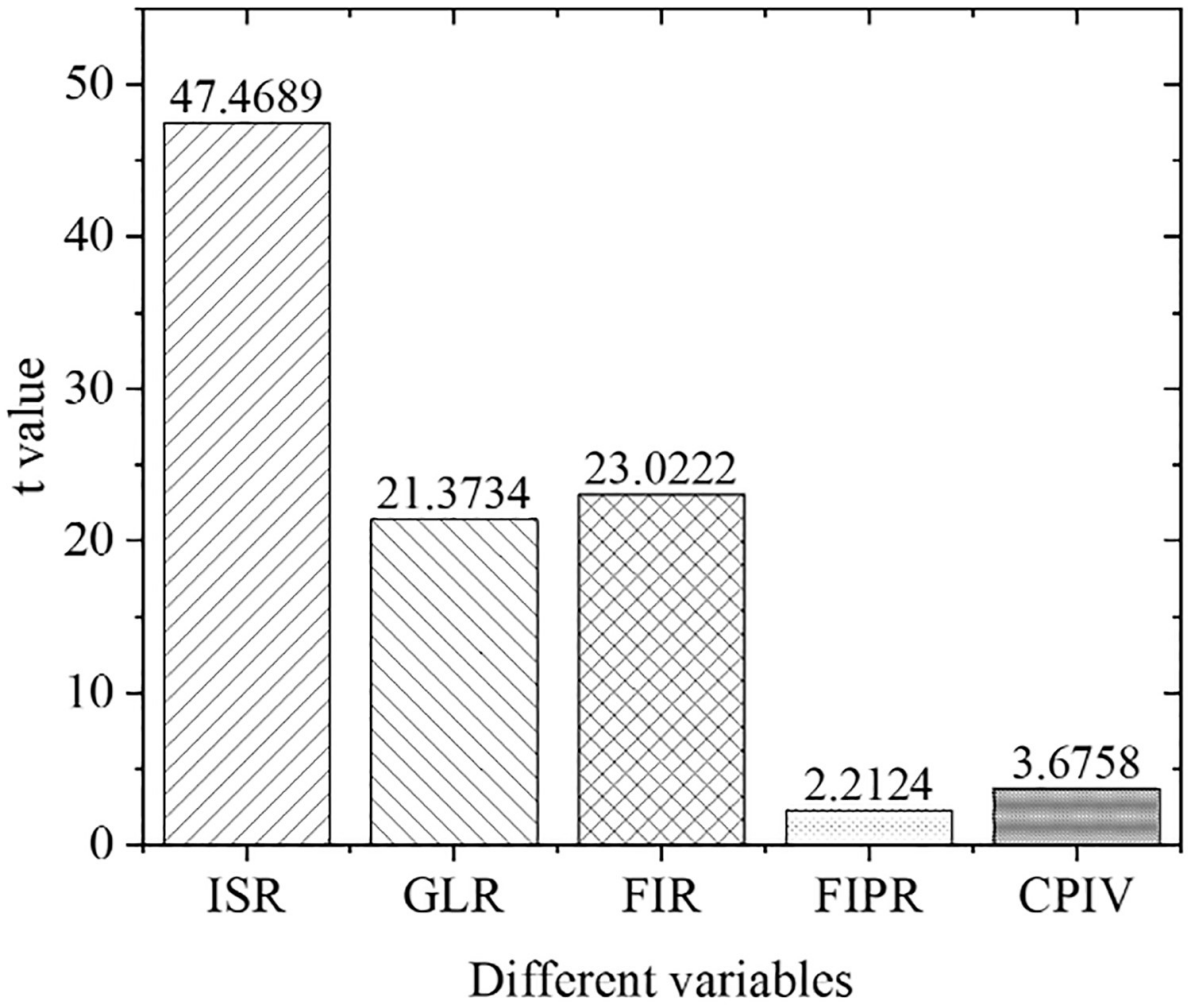
Calculation results of overall t value.

Fig 5 shows the estimation results of the overall empirical panel coefficient, and Fig 6 displays the calculation results of the overall t-value. An increase in the financial interrelations ratio will significantly improve the industrial structure optimization rate, indicating that the increase in the financial interrelations ratio will drive the industrial structure optimization, with significant influences. The coefficient of the financial correlation rate is positive, indicating that the industrial structure and financial development level have a significant positive correlation. The coefficient of the financial industry product ratio is positive. Therefore, as the financial industry production ratio increases, the industrial structure optimization rate will increase significantly, indicating that the continuous expansion of the financial industry as the tertiary industry will definitely bring about a continuous increase in the proportion of the tertiary industry. As a result, the optimization and upgrading of China’s industrial structure will be promoted. With the increase in policy credit intervention, the rate of industrial structural optimization has increased significantly, proving the effects of government credit intervention.

### 4.4. Regional estimation analysis

**Fig 7:**
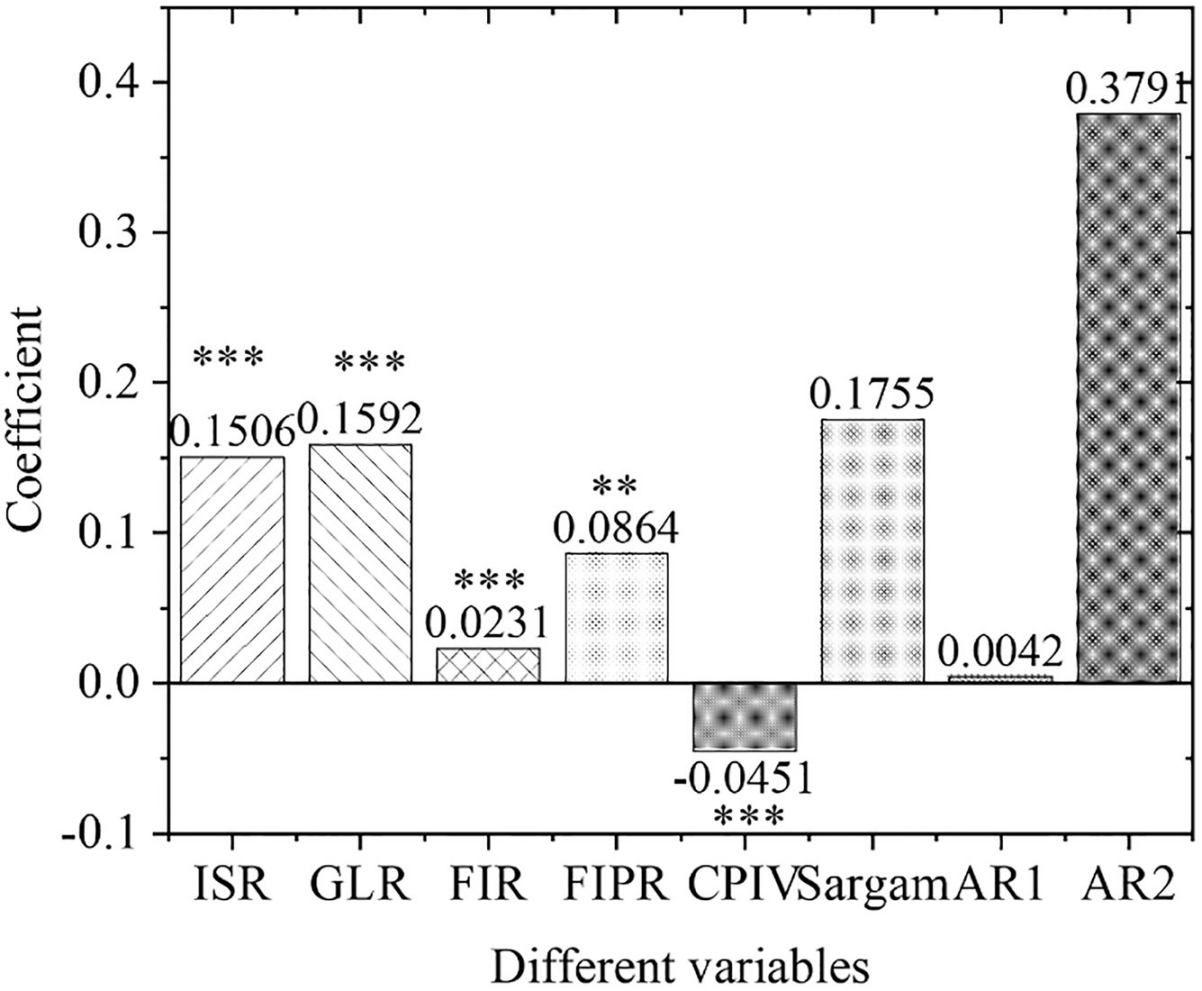
Estimation results of empirical panel coefficient in eastern China.

**Fig 8:**
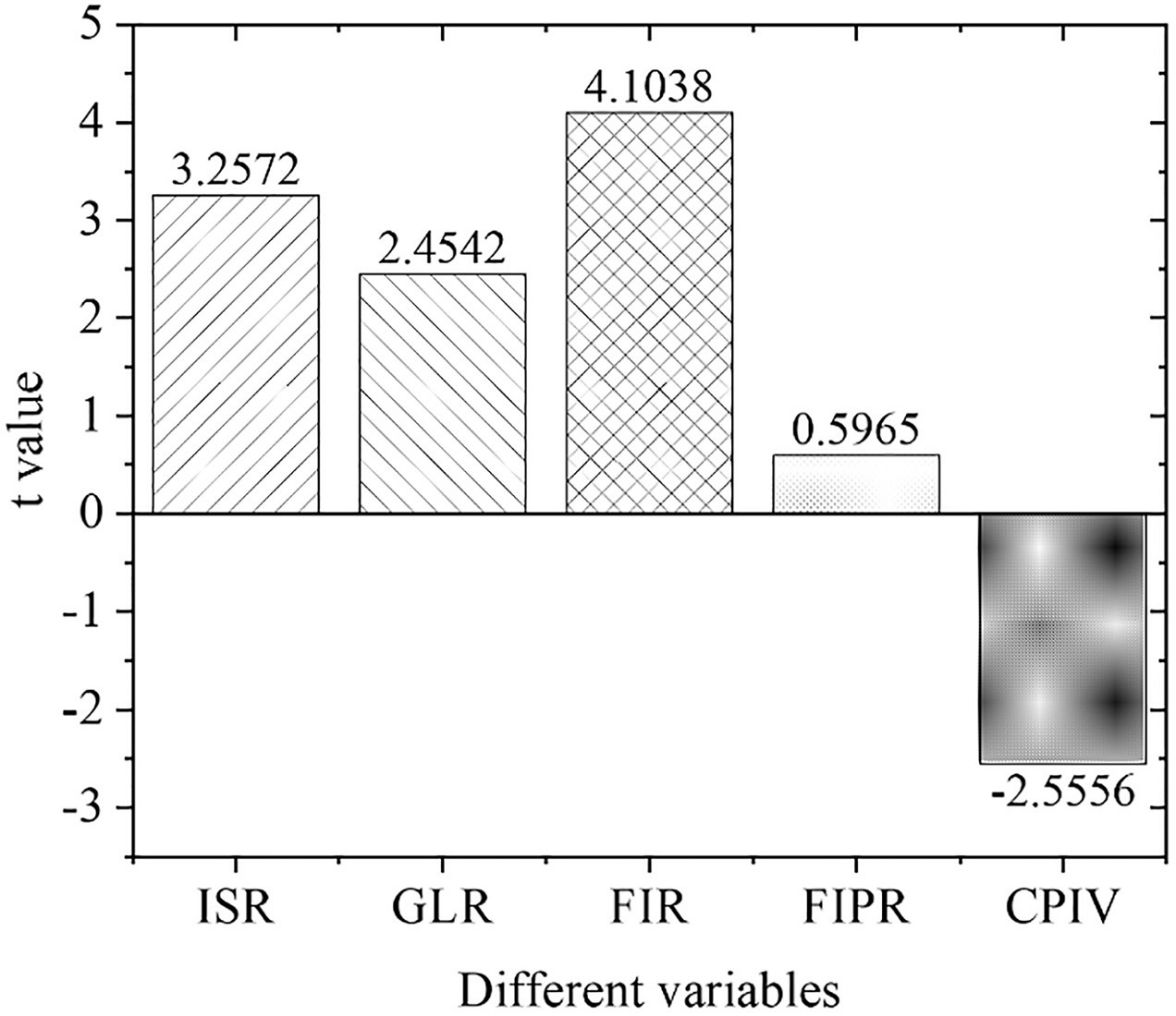
Calculation results of t value in the eastern region.

Fig 7 demonstrates the results of empirical panel coefficient estimation results in the eastern region, and Fig 8 presents the *t*-value calculation results in the eastern region. As far as the eastern region is concerned, the industrial structure adjustment and upgrading, the financial development level, and the green credit development are strong influencing factors that promote the current industrial structure optimization and upgrading. The supply of green credit significantly impacts the industrial structure adjustment that year, which is 0.35% higher than the overall national level, indicating that the green credit can significantly promote industrial structure optimization and upgrading in the eastern region. The coefficient of the financial interrelations ratio is positive, indicating that the industrial structure upgrading in the eastern region has a significantly positive correlation with the level of financial development. The increase in the financial interrelations ratio will significantly improve the rate of industrial structural optimization, indicating that the increase in financial development in the eastern region positively affects the upgrading of industrial structure.

**Fig 9:**
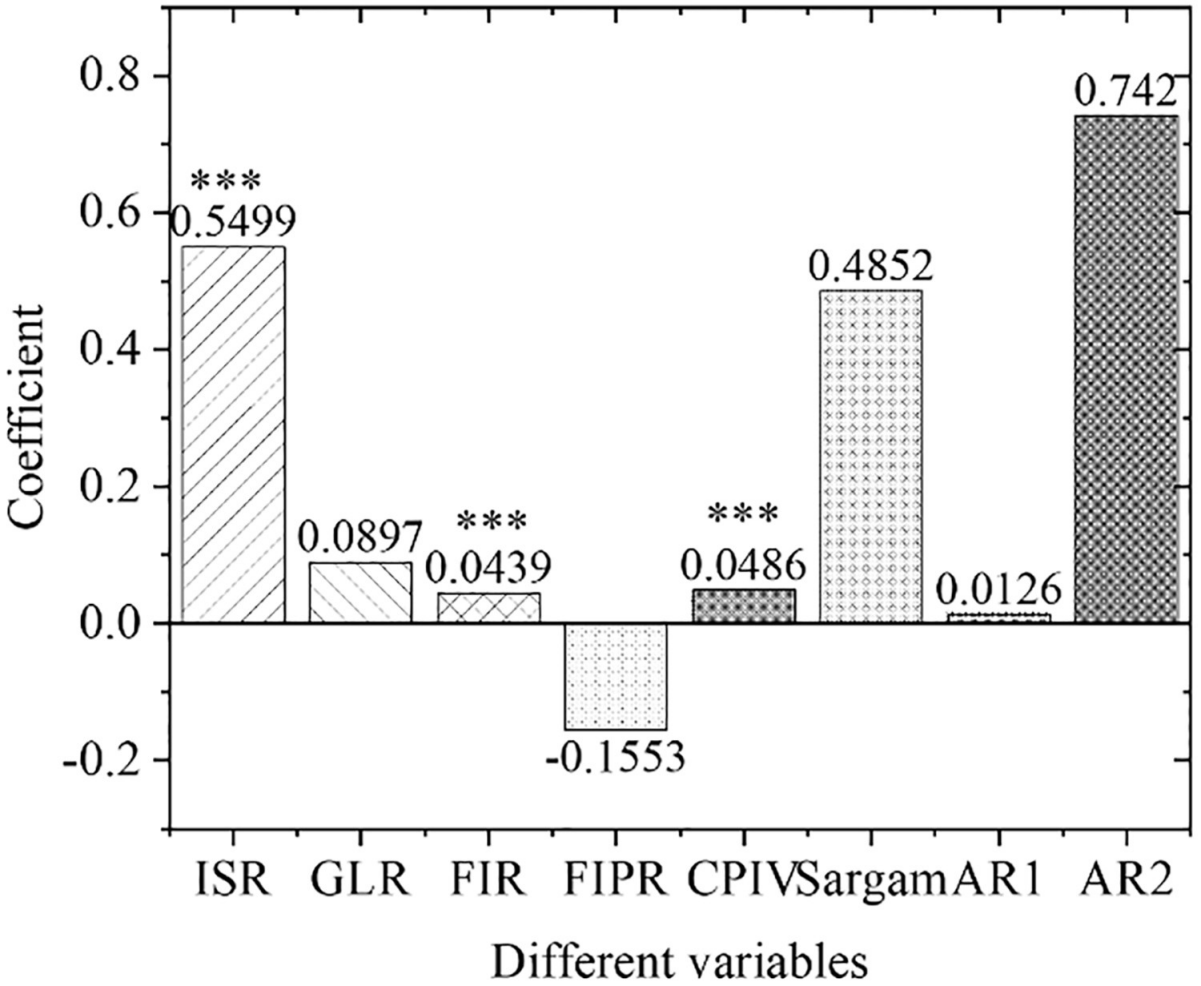
Estimation results of empirical panel coefficients in Central China.

**Fig 10:**
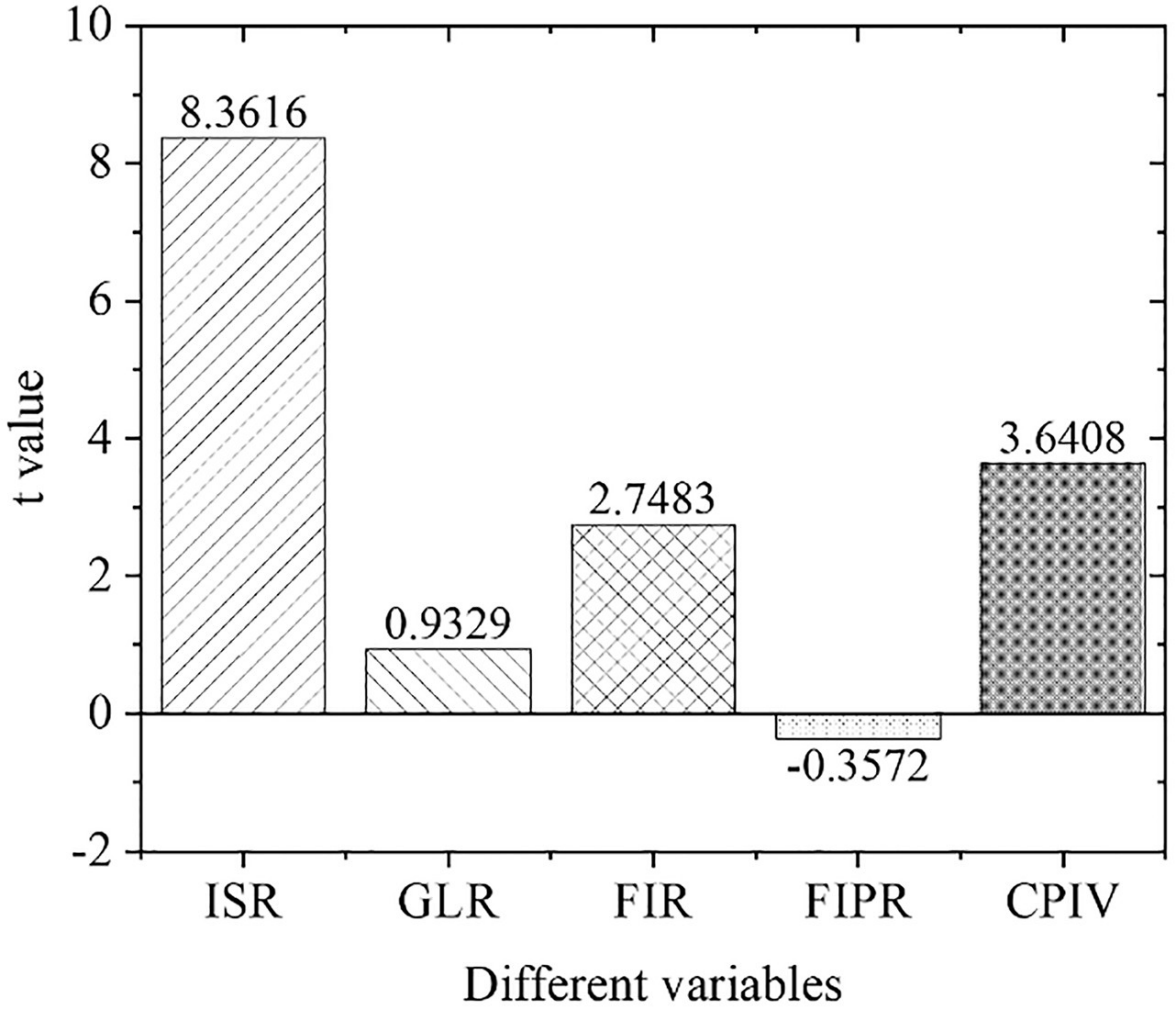
Calculation results of t value in Central China.

Fig 9 displays the estimation results of the empirical panel coefficients of the central region, and Fig 10 shows the calculation results of the *t*-value of the central region. As far as the central region is concerned, the development of its green credit business and the scale of financial industry development have not been prominent in promoting the upgrading of regional industrial structural optimization. The economic and financial problems in the central region have caused problems with the original industrial structure. In the meantime, the allocation of funds is not perfect, the capital market is immature, the original enterprises have fewer capital sources for environmental protection transformation and upgrading, and the emerging environmental protection enterprises also lack quality improvement and independent innovation abilities.

**Fig 11:**
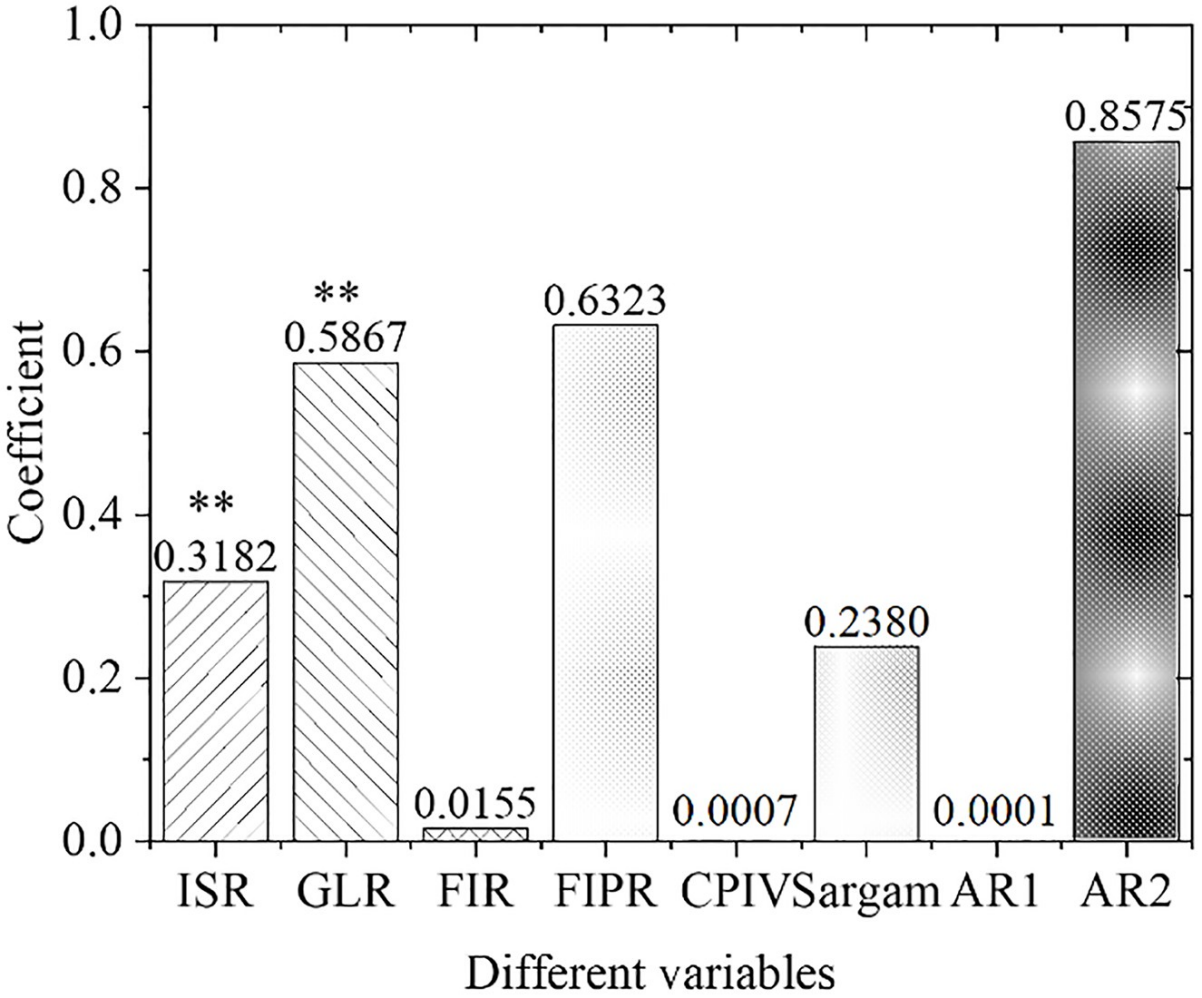
Estimation results of empirical panel coefficient in Western China.

**Fig 12:**
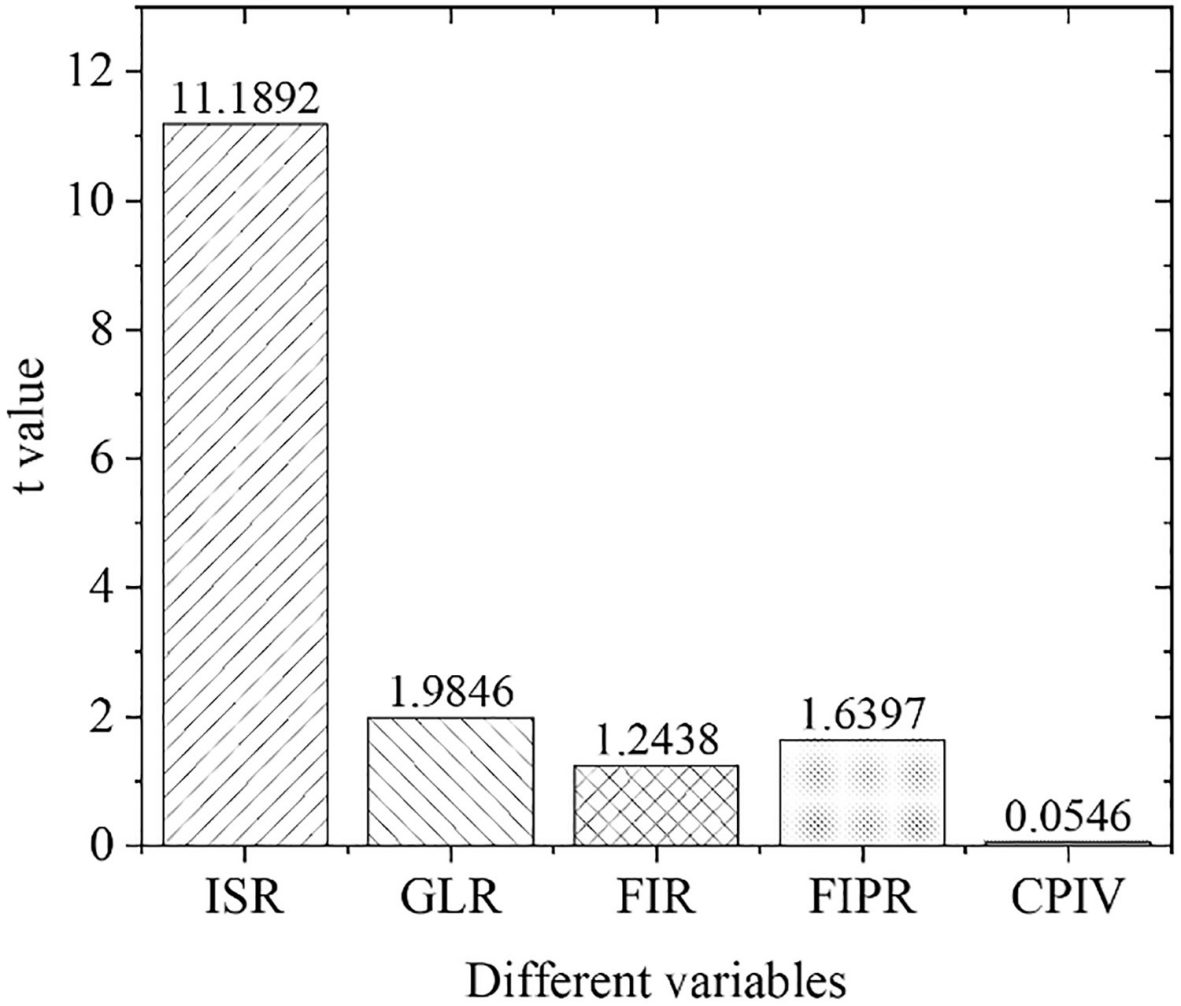
Calculation results of t value in Western China.

Fig 11 presents the estimation results of the empirical panel coefficients in the western region, and Fig 12 illustrates the calculation results of the *t*-value in the western region. As far as the western region is concerned, the industrial structure adjustment and the current level of financial development positively affect the adjustment of the current industrial structure, indicating that the green credit policy has played a promotive role in the industrial structure adjustment of this region. The financial industry product and the role of government credit policies in promoting the upgrading of regional industrial structural optimization have not been prominent in the western region. The coefficient of financial interrelations ratio is positive, indicating a significant positive correlation between the industrial structure and the supply of green credit. In other words, as the economy develops and the supply of green credit continues to increase, the industrial structure will continue to be optimized and adjusted; in the meantime, the primary industry’s share of GDP will gradually decrease, while the proportions of the secondary and tertiary industries in GDP will increase.

### 4.5. Robustness analysis

**Fig 13:**
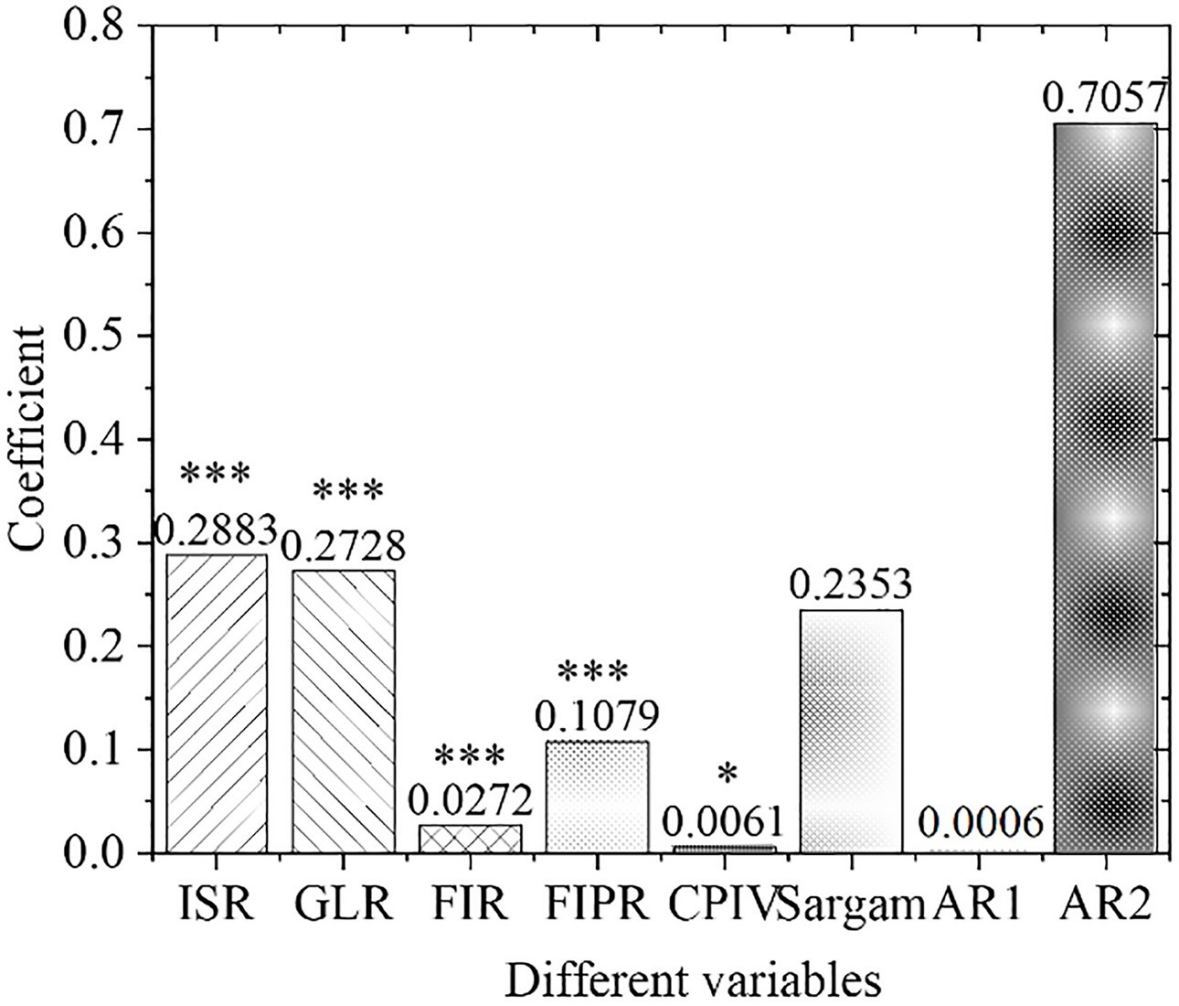
Robustness analysis results (empirical panel coefficient estimation of the whole country).

**Fig 14:**
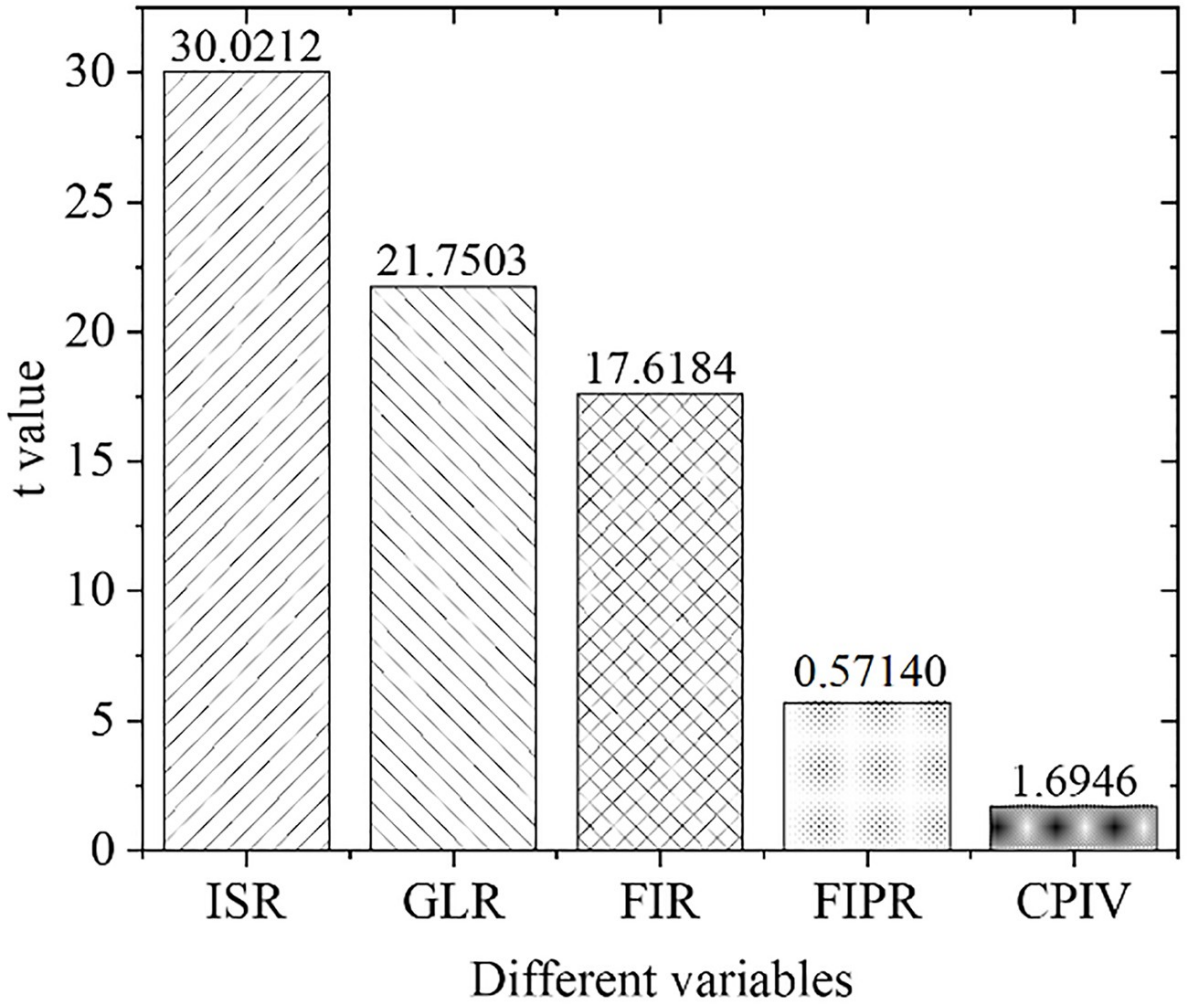
Robustness analysis results (t-value of the whole country).

**Fig 15:**
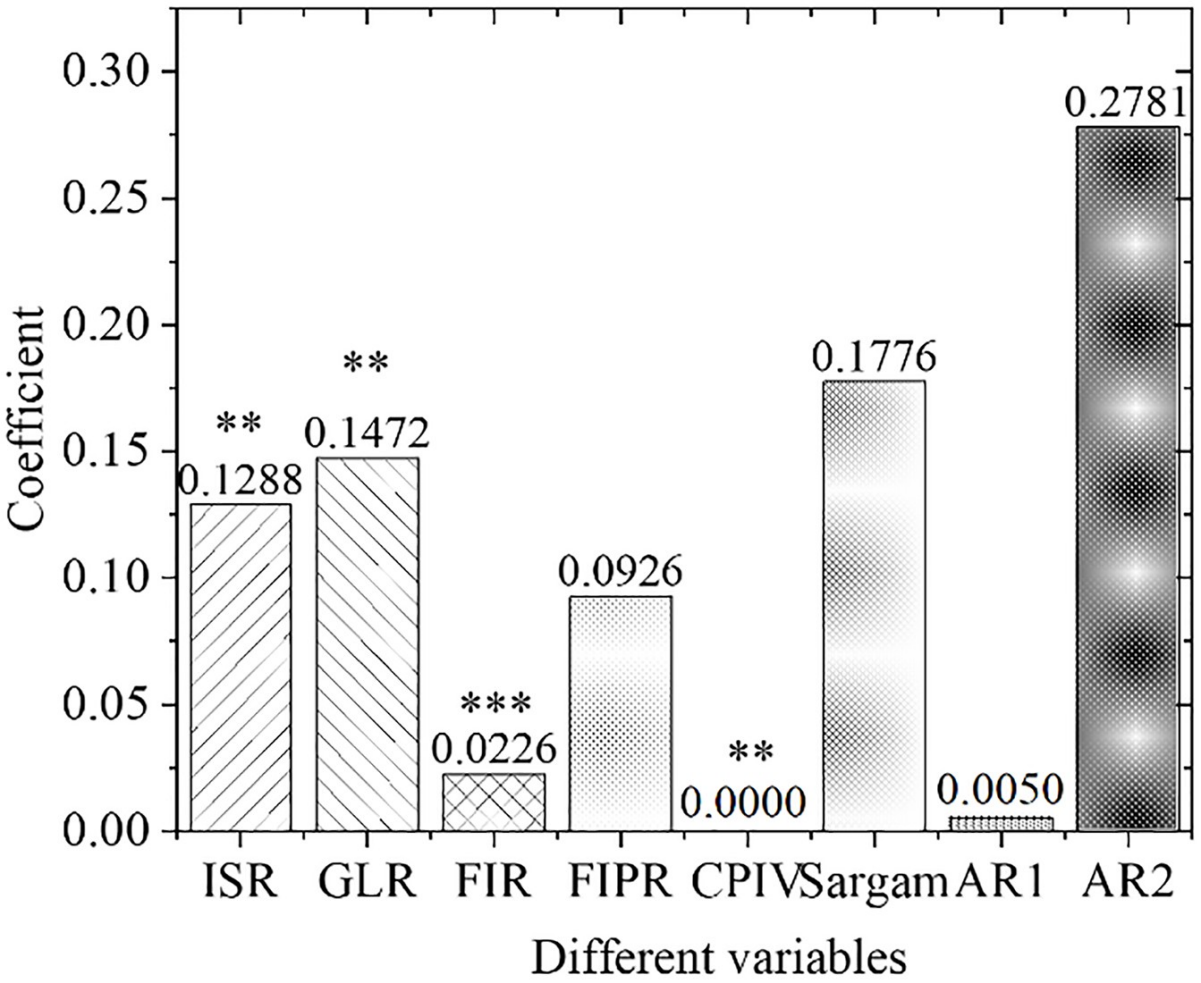
Robustness analysis results (empirical panel coefficient estimation in the eastern region).

**Fig 16:**
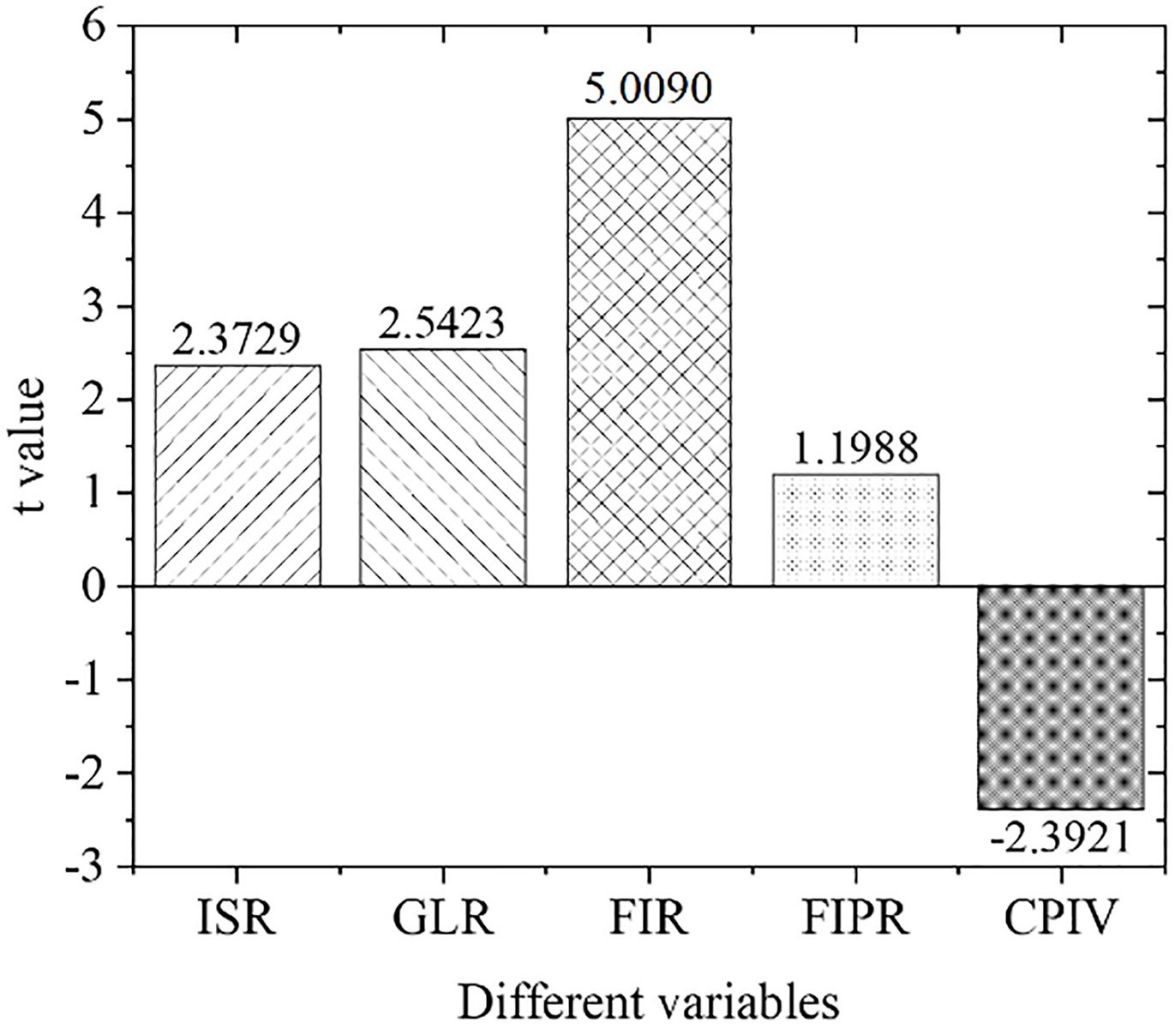
Robustness analysis results (t-value in the eastern region).

Figs 13 to 16 show the empirical panel coefficient estimates and *t*-value results for the whole country and the eastern region, respectively. In the original model, there are basically no alternative indicators for the independent variable, that is, green credit. Therefore, the variable financial industry product ratio is substituted with the regional financial industry product’s share of the regional tertiary industry to measure the model regression robustness. The significances of the principal explanatory variable (that is, the financial interrelations ratio) and the structural optimization level of the previous period have basically no changes, and their influence degrees are similar. The coefficient signs and significance levels of other variables have not changed significantly, indicating that the regression model based on dynamic panel data has better robustness.

## 5. Conclusions

The green credit business is taken as the research object. Based on previous research, the relation between the green credit business and the industrial structure is analyzed. A grayscale analysis model is constructed based on the GRA theory, and the relation between China’s financial interrelations ratio and the industrial structure is calculated. The national overall panel regression analysis and regional panel analysis models are established using China’s provincial panel data from 2009 to 2019. Moreover, the structure of the model is optimized through the trading structure to understand the changes in the industrial structure in China. Green credit has affected China’s industrial structural optimization adjustments from capital formation, signal transmission, and feedback credit. It affects the industrial structure through enterprises’ capital and funding channels. China’s overall green credit adjustment significantly affects the industrial structure adjustment. The influence of green credit on the industrial structure adjustment differs in the three major regions of the East, Central, and West. A suitable analysis model has been constructed; nevertheless, several shortcomings are found in it. First, the GRA model is adopted, while the latest neural network and deep learning algorithms are not involved in data processing, which may reduce the prediction accuracy of the model. Second, factors affecting the green credit business are found; however, their roles in the financial field are not analyzed in-depth. Hence, these two aspects will be explored and researched in-depth in the future to improve the analysis model, to provide a theoretical basis for the green development of China’s follow-up financial market and the application of deep learning neural network algorithm under the background of IoT.

## Supporting information

S1 Data (XLSX)
